# Designing Experiments to Discriminate Families of Logic Models

**DOI:** 10.3389/fbioe.2015.00131

**Published:** 2015-09-04

**Authors:** Santiago Videla, Irina Konokotina, Leonidas G. Alexopoulos, Julio Saez-Rodriguez, Torsten Schaub, Anne Siegel, Carito Guziolowski

**Affiliations:** ^1^UMR 6074 IRISA, CNRS, Campus de Beaulieu, Rennes, France; ^2^Dyliss project, INRIA, Campus de Beaulieu, Rennes, France; ^3^Institut für Informatik, Universität Potsdam, Potsdam, Germany; ^4^LBSI, Fundación Instituto Leloir, CONICET, Buenos Aires, Argentina; ^5^IRCCyN UMR CNRS 6597, École Centrale de Nantes, Nantes, France; ^6^Department of Mechanical Engineering, National Technical University of Athens, Athens, Greece; ^7^European Molecular Biology Laboratory, European Bioinformatics Institute, Hinxton, UK

**Keywords:** experimental design, Boolean logic models, phosphoproteomic, answer set programming, signaling networks

## Abstract

Logic models of signaling pathways are a promising way of building effective *in silico* functional models of a cell, in particular of signaling pathways. The automated learning of Boolean logic models describing signaling pathways can be achieved by training to phosphoproteomics data, which is particularly useful if it is measured upon different combinations of perturbations in a high-throughput fashion. However, in practice, the number and type of allowed perturbations are not exhaustive. Moreover, experimental data are unavoidably subjected to noise. As a result, the learning process results in a family of feasible logical networks rather than in a single model. This family is composed of logic models implementing different internal wirings for the system and therefore the predictions of experiments from this family may present a significant level of variability, and hence uncertainty. In this paper, we introduce a method based on Answer Set Programming to propose an optimal experimental design that aims to narrow down the variability (in terms of input–output behaviors) within families of logical models learned from experimental data. We study how the fitness with respect to the data can be improved after an optimal selection of signaling perturbations and how we learn optimal logic models with minimal number of experiments. The methods are applied on signaling pathways in human liver cells and phosphoproteomics experimental data. Using 25% of the experiments, we obtained logical models with fitness scores (mean square error) 15% close to the ones obtained using all experiments, illustrating the impact that our approach can have on the design of experiments for efficient model calibration.

## 1 Introduction

The recent development of high-throughput experimental technologies allows us to observe different cellular parts under multiple situations. This information is of great value to generate and validate computational models of the molecular processes happening within cells.

Thanks to their simplicity, qualitative approaches allow us to model larger-scale biological systems than quantitative methods. Among these approaches, logic models are able to capture interesting and relevant behaviors in the cell (Morris et al., 2010; Mbodj et al., 2013). We have previously proposed to generate logic models by training a prior knowledge network to phosphoproteomics data. Importantly, due to factors, such as the sparsity and the uncertainty of experimental measurements, there are often multiple models that cannot be distinguished with the data at hand, that is, the model is non-identifiable, requiring to consider a set (a family) of logic models (Saez-Rodriguez et al., 2009; Guziolowski et al., 2013). In this paper, we propose to specialize the (possibly many) logic behaviors of this family by using an efficient strategy for experiment design, that is, an optimal selection of signaling perturbations to discriminate models at hand.

The experimental design problem consists of finding the most informative experiments in order to identify more accurate models (Kreutz and Timmer, 2009). On the one hand, in the context of quantitative models, this problem has been approached via methods for both parameter estimation and model discrimination (Kremling et al., 2004; Vatcheva et al., 2005; Mélykúti et al., 2010; Busetto et al., 2013; Stegmaier et al., 2013; Meyer et al., 2014). On the other hand, for qualitative models, fewer methods have been proposed (Ideker et al., 2000; Yeang et al., 2005; Barrett and Palsson, 2006; Szczurek et al., 2008; Sparkes et al., 2010; Atias et al., 2014). An optimal experimental design can be applied to either: (*i*) experimental setup selection, that is the optimal choice of species to perturb and measure, or (*ii*) perturbations selection, where one perturbation indicates which species will be perturbed in one experiment.

In this work, we focus on experimental design for selecting an optimal signaling perturbation set by considering the experimental setup fixed and a set of initial measurements from low combinatorial (i.e., single stimulus or inhibitor species) perturbations. We use training algorithms to identify a family of Boolean models explaining the data according to a prior knowledge network. This family needs to be discriminated by measuring the effect of additional perturbations. For this, we propose a new method that finds optimal set of signaling perturbations satisfying the following criteria: (*i*) it contains a minimal number of perturbations to discriminate all pairs of models in a family of Boolean networks, (*ii*) such perturbations maximize the pairwise differences of models’ predictions, and (*iii*) they are subject to technologically inspired constraints, such as the minimization of experimental perturbations cost.

Compared to previous contributions in the context of logical models, our work presents certain differences and similarities. In general, in previous methods, the optimality criterion for a selection of perturbations is given by means of the so-called Shannon entropy (Shannon, 1948). In this context, the Shannon entropy provides a measure of the expected information gained in performing a specific experiment. Intuitively, the higher the Shannon entropy, the higher the ability of an experimental perturbation to distinguish between rival models (Ideker et al., 2000; Szczurek et al., 2008; Atias et al., 2014). In contrast, in our work, the main optimality criterion consists of maximizing the sum of pairwise differences over Boolean models’ output. The intuition behind this criterion is to increase the chances to discriminate a pair of models despite the experimental noise. Nonetheless, it is worth noting that some pairs of models could be better discriminated than others. Thus, in principle, if one aims at having a more uniform pairwise discrimination, an entropy-based design criterion would be more appropriate. However, approaches based on the Shannon entropy must resign to exhaustiveness due to computational scalability. Maximizing the sum of pairwise differences was already proposed by Mélykúti et al. (2010) for the discrimination of ODEs models and, recently, the same idea has been used in the context of Boolean logic models (Atias et al., 2014). However, in contrast to our approach, the method introduced by Atias et al. (2014) aims at finding only one perturbation maximizing the number of differences in the output of the pair of models, which differs the most from each other. Therefore, in general, it does not guarantee that other pairs of models will be discriminated as well. More generally, except for Szczurek et al. (2008), previous approaches proposed assays composed of one perturbation. Therefore, only after the proposed perturbation has been carried out in the laboratory and models have been (partially) discriminated, another perturbation can be designed. In contrast, but similarly to Szczurek et al. (2008), we find the smallest number of perturbations to optimally discriminate all pairs of models at once. This approach is tailored to high-throughput technologies that are designed to measure the effects of tens of perturbations in a single run.

We provide a precise characterization of the combinatorial problem related to the optimal selection of signaling perturbations, together with an Answer Set Programming (Gebser et al., 2012) based solution to this problem included within the open source python package **caspo**, which is freely available for download^1^. We applied our method to two case studies using *in silico* and real phosphoproteomics datasets to measure the impact of our approach in a real setting. We show that optimal logic models with few input–output behaviors can be learned by combining a set of low combinatorial perturbations with a minimal set of greater combinatorial perturbations. In the artificially generated data, we obtained that phosphoproteomics measurements from 64 low combinatorial perturbations can be enriched with 10 combinatorial perturbations (from the 1630 possible) to identify a family of logic models with a fitness quality (mean square error) equal to one of the golden standard logic model used to generate the data. In the real dataset, we obtained that phosphoproteomics measurements from 12 low combinatorial perturbations can be enriched with 31 combinatorial perturbations (from the 120 available) to identify a family of logic models with a fitness quality at a 15% distance from the fitness of logical models explaining optimally all 120 responses to the perturbations considered.

## 2 Materials and Methods

### 2.1 Background

In this paper, we are interested in the discrimination of models based on synchronous Boolean networks (Kauffman, 1969). Importantly, we restrict ourselves to BNs describing models of immediate-early response as introduced in Saez-Rodriguez et al. (2009). Since we focus on fast (early) events, it is assumed that oscillation or multi-stability caused by feedback-loops (Remy et al., 2008) cannot happen until a second phase of signal propagation occurring at a slower time scale. Therefore, BNs with feedback-loops are not considered (Macnamara et al., 2012).

Several related methods within this framework were published in the last few years in order to learn BNs from a prior knowledge network (PKN) and a phosphoproteomics dataset (Mitsos et al., 2009; Saez-Rodriguez et al., 2009; Guziolowski et al., 2013; Sharan and Karp, 2013; Videla et al., 2014). A PKN is a signed and directed graph describing causal relations among a set *V* of nodes representing biological species. An *experimental setup* is defined by three subsets of *V*, namely, possible *stimuli* (*V_S_*), possible *inhibitors* (*V_I_*), and *measured* species (*V_M_*). A *signaling perturbation* is a combination of present/absent stimuli and inhibitors. Then, a phosphoproteomics dataset provides phosphorylation activities (in this context, immediate-early responses) of a set of measured species or readouts under several signaling perturbations. Note that any signaling perturbation is described by an *n*-dimensional Boolean vector, i.e., *p* ∈ B^*n*^, where *n* = |*V_S_*| + |*V_I_*| and B = {0,1}. More precisely, if the *j^th^* position in *p* is assigned to 1 (resp. 0), the corresponding stimulus or inhibitor is said to be present (resp. absent) in the experimental perturbation *p*.

In general, aforecited methods for learning BNs explore the space of models compatible with the topology given by the PKN aiming at the minimization of two criteria, namely, the difference between data and model predictions, and the model size. On the one hand, the difference between data and model predictions is measured by means of the Mean Squared Error (MSE). On the other hand, the size of a BN is defined as the sum of its formulas’ length. Further, due to the inherent noise in experimental data, we are interested not only on optimal but also nearly optimal BNs. That is, BNs having MSE and size within given tolerances with respect to the corresponding minimal values. In this context, it has been shown that the exhaustive enumeration of nearly optimal BNs explaining phosphoproteomics dataset with respect to a PKN leads to a large number of them (Guziolowski et al., 2013). Nonetheless, it often happens that for all measured species, several BNs describe exactly the same response to every possible signaling perturbation. In such a case, we say that those BNs describe the same *input–output behavior*. For example, in Guziolowski et al. (2013), several thousands of nearly optimal BNs described only 91 distinct responses. Concretely, the input–output behavior of a BN is described by a “truth table” whose entries are all possible signaling perturbations, and whose outputs are the corresponding Boolean vector responses. Therefore, we can see input–output behaviors merely as functions of the form β: B^*n*^ → B^*m*^ where *n* = |*V_S_|* + |*V_I_*| and *m* = |*V_M_*|. Notice that, for a given set of BNs, we can identify a canonical set of input–output behaviors ***B*** containing exactly one *representative* BN for each behavior.

### 2.2 Discriminating input–output behaviors

In this section, we introduce our method to discriminate input–output behaviors in a pairwise fashion. We assume that we are given a set ***B*** of input–output behaviors. For instance, this set may result from the learning of BNs from given PKN and phosphoproteomics dataset. In what follows, we denote by ***D*** a selection of signaling perturbations. In theory, all combinatorial perturbations of stimuli and inhibitors could be considered. Nonetheless, in order to consider the limitation of current technology, in general, we restrict ourselves to perturbations having at most *s* stimuli and *i* inhibitors, with 0 ≤ *s* ≤ |*V_S_|* and 0 ≤ *i* ≤ |*V_I_*|. Then, we denote with ***P*** the set of such possible signaling perturbations. Notably, the total number of perturbations is given by:

$$\sum_{j=0}^{s}\;\left(\begin{array}{l} \left|V_{S}\right|\\ j \end{array}\right)\times \sum_{j=0}^{i}\;\left(\begin{array}{l} \left|V_{I}\right|\\ j \end{array}\right)$$

where nm denotes the binomial coefficient, i.e., $\frac{n!}{m!\left(n-m\right)!}$. Alternatively, ***P*** could be defined by the user providing any fixed list of feasible perturbations. Next, our method has three main steps: (1) find the minimum number *k* of signaling perturbations in ***P*** to discriminate every pair of input–output behaviors in ***B***, (2) find all sets of exactly *k* signaling perturbations in ***P*** maximizing the sum of pairwise differences of input–output behaviors in ***B***, and (3) minimize the complexity of the experiments by minimizing the number of present stimuli and inhibitors in the set of selected perturbations. In what follows, we give more details and mathematical definitions for each of these steps. In addition, we introduce a parameter *k_max_* describing the maximum number of perturbations that can be performed simultaneously.

#### 2.2.1 Step 1: Required Signaling Perturbations to Discriminate all Behaviors Pairwise

Usually, several perturbations must be performed in order to discriminate among every pair of behaviors. However, in order to minimize experimental costs, one would like to perform as few perturbations as possible. Therefore, our first criterion consists of finding the minimum number of perturbations, which allow us to discriminate among every pair of input–output behaviors. To be more precise, we aim at finding the smallest *k* ∈ (0, *k_max_*) such that there exists a set ***D*** having *k* perturbations *p* ∈ ***P*** satisfying:

(1) $$\left(\forall \beta ,\beta^{\prime }\in B::\left(\exists p\in D::\beta \left(p\right)\neq \beta^{\prime }\left(p\right)\right)\right).$$

Let us denote with D_(_*_k,s,i_*_)_ the set of all ***D*** ⊆ ***P*** with |***D***| = *k* and satisfying (1). It is worth noting that we restrict our search to at most *s* stimuli, *i* inhibitors, and *k_max_* perturbations. Therefore, there may be cases where does not exists ***D*** discriminating all input–output behaviors pairwise. For such cases, we relax the constraint of full pairwise discrimination and define D_(_*_k,s,i_*_)_ as before but setting *k* = *k_max_* and without requiring the satisfaction of (1). That is, some but not all pairs of input–output behaviors are discriminated.

#### 2.2.2 Step 2: Maximizing Differences Over Measured Species

Once we have identified that *k* signaling perturbations are required to discriminate between all input–output behaviors in ***B*** (or alternatively, *k* = *k_max_*), the next question is how to select among all possible sets ***D*** ∈ D_(_*_k,s,i_*_)_. Then, we define the *differences* (Θ*_diff_*) generated by a set ***D*** ∈ D_(_*_k,s,i_*_)_ over the family of input–output behaviors ***B*** as:

(2) $$\Theta_{\mathrm{diff}}\;\left(B,D\right)=\sum_{\beta ,\beta^{\prime }\in B}\sum_{p\in D}H\left(\beta \left(p\right),\beta \prime \left(p\right)\right)$$

where H denotes the Hamming distance over Boolean vectors, i.e., the number of positions at which the corresponding vectors values are different. Our second criterion consists of finding all sets of *k* perturbations ***D*** ∈ D_(_*_k,s,i_*_)_ such that the function Θ*_diff_* is maximized,

(3) $$D_{\left(k,s,i\right)}^{*}=\underset{D\in D_{\left(k,s,i\right)}}{\arg\;\max}\;\Theta_{\mathrm{diff}}\;\left(B,D\right).$$

#### 2.2.3 Step 3: Minimizing the Complexity of Experiments

The complexity of any signaling perturbation is essentially related to the number of present stimuli and inhibitors in it. Thus, in this step, we aim at finding the simplest sets of perturbations among all $D^{*}\in D_{\left(k,s,i\right)}^{*}$. Toward this end, we define two functions counting the number of stimuli ($\Theta_{V_{S}}$) and inhibitors ($\Theta_{V_{I}}$) being present in a given set of signaling perturbations. More precisely, let us recall that every perturbation *p* is a Boolean vector such that, if the *j^th^* position in *p* is assigned to 1 (resp. 0), the corresponding stimulus or inhibitor is said to be present (resp. absent) in *p*. Thus, for the set *U* = *V_S_* or *U* = *V_I_* of either stimuli or inhibitors, we can define Θ*_U_* as,

(4) $$\forall D^{*}\in D_{\left(k,s,i\right)}^{*},\;\Theta_{U}\left(D^{*}\right)=\sum_{p\in D*}\sum_{u_{j}\in U}p_{j}$$

where *p_j_* denotes the *j^th^* position in *p* corresponding to either a stimulus if *U* = *V_S_*, or an inhibitor if *U* = *V_I_*. Finally, we consider two additional optimization criteria in lexicographic order (Marler and Arora, 2004) aiming at the identification of the simplest $D^{*}\in D_{\left(k,s,i\right)}^{*}$, and we define the family of optimal sets of signaling perturbations ***D_opt_*** ∈ D_*opt*_ as follows:

(5) $$D_{\mathrm{opt}}=\underset{D^{*}\in D_{\left(k,s,i\right)}^{*}}{\arg\;\min}\left(\Theta_{V_{S}}\left(D^{*}\right),\Theta_{V_{I}}\;\left(D^{*}\right)\right).$$

Notice that we minimize first $\Theta_{V_{S}}$ and then, with lower priority $\Theta_{V_{I}}$, but this is an arbitrary choice, which can be revisited.

### 2.3 Experimental design powered by answer set programming

The method described in Section 2 is implemented in the publicly available python package **caspo**. Our software strongly relies on a form of logic programming known as Answer Set Programming (ASP) (Gebser et al., 2012). ASP provides a declarative framework for modeling knowledge-intense combinatorial (optimization) problems. Moreover, state-of-the-art ASP solvers offer powerful implementations. In our context, the ASP logic program is satisfiable for positive integers *k, s, i* if there exists a selection of *k* signaling perturbations in ***P*** satisfying (1). Then, the solving consists of considering, starting from *k* = 1, increasing values for *k* until the ASP logic program is satisfiable. Once the solver finds the smallest *k* or reaches *k_max_*, it proceeds to solve the multi-objective optimization problem in lexicographic order: first, by maximizing the pairwise differences over the Boolean models’ outputs as defined in (3), and then by minimizing the complexity of experimental perturbations, as defined in (5). It is worth noting that, thanks to the declarativeness and elaboration tolerance of ASP, it is straightforward to consider additional constraints for specific use cases.

### 2.4 The loop for learning and discriminating input–output behaviors

**Figure 1:**
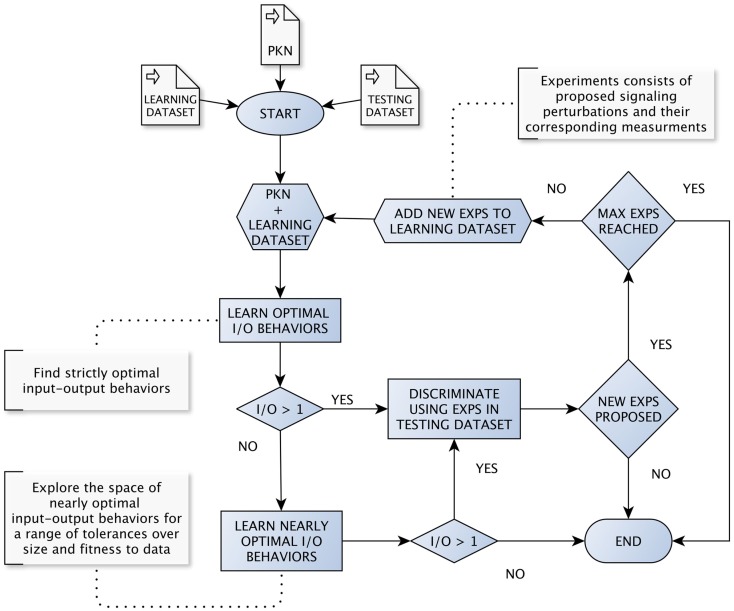
**The loop for learning and discriminating input–output behaviors**. The loop starts by learning optimal input–output behaviors from a given PKN and initial dataset. Then, we try to discriminate among learned input–output behaviors as soon as we find more than one. Every time we discriminate among a set of behaviors, an optimal set of signaling perturbations is proposed. Next, both the set of perturbations and the corresponding measurements are added to the dataset used for learning and the loop starts over. When the learning method returns a single optimal input–output behavior, the workflow explores nearly optimal behaviors by considering a range of tolerances, first over the optimum model size and then over the optimum MSE.

In what follows we assume the existence of a method for learning nearly optimal BNs and their corresponding set of input–output behaviors. Further, such a method must be parametrized specifying allowed tolerances with respect to optimal fitness and size. Also, we assume an implementation of the method described in Section 2. In our case, we rely on the python package **caspo**, which implements both methods providing an unified framework. In order to evaluate our method in a systematic way, we have implemented the workflow shown in Figure 1,^2^. For a more detailed description, we refer the reader to pseudo-code algorithms (Algorithm S1 and S2) provided in Supplementary Material.

We start by learning strictly optimal input–output behaviors from a given PKN and initial dataset. Then, the workflow follows a “cautious” strategy in the sense that it will try to discriminate among input–output behaviors as soon as we find more than one. Every time we discriminate among a set of input–output behaviors, an optimal set of signaling perturbations is proposed. Then, both the set of perturbations and the corresponding measurements obtained after performing the experiments are added to the dataset used for learning and the workflow starts over. Notably, in our simulations, measurements are extracted automatically from either artificial or real datasets available beforehand. Meanwhile, in real case studies, measurements would be provided by concrete wet experiments.

Importantly, there are cases when the learning method returns a single optimal input–output behavior. In such cases, the workflow explores nearly optimal behaviors by considering a range of tolerances, first over the optimal model size and then over the optimal MSE. Extending the discrimination procedure to nearly optimal behaviors allows ensuring that the complete workflow is robust to noise in data. Nonetheless, after considering certain ranges of tolerances on both size and fitness to data, there could be only one input–output behavior. In such a case, we interpret the behavior at hand to be robust enough and the workflow terminates. Otherwise, the workflow has two additional stop conditions: (1) when all proposed signaling perturbations for discrimination are already present in the dataset used for learning; (2) when the number of experiments in the dataset reaches a given maximum number of allowed experiments.

## 3 Results

### 3.1 Experimental design on artificial and real case studies

We evaluate our approach using the workflow described in Section 4 for real-world signaling pathways in human liver cells, and both artificial and real phosphoproteomics datasets. At every loop iteration, we compute two metrics over the learned input–output behaviors: (1) the *learning* MSE, which is computed with respect to the dataset used for learning, and (2) the *testing* MSE, which is computed with respect to the complete space of signaling perturbations under consideration (either artificial or real datasets available beforehand).

#### 3.1.1 Artificial Case Studies

The PKN was introduced in Saez-Rodriguez et al. (2009) and here we use a variation that we used also in Guziolowski et al. (2013). Further, to motivate our study, we considered the experimental setup (choice of stimuli, inhibitors, and measured species) from a publicly available phosphoproteomics dataset (Alexopoulos et al., 2010). It contains 7 stimuli, 7 inhibitors, and 15 readouts. Using the PKN, we have generated 100 random BNs as our *gold standards*. We require that every gold standard has size between 28 and 32, and between 2 and 4 AND gates. Next, for each gold standard, an artificial Boolean dataset is generated by performing the simulation of every possible signaling perturbation over the network. That is, each artificial dataset consists of 2^14^ signaling perturbations with their corresponding output measurements. Moreover, toward more realistic phosphoproteomics datasets, we add random noise to Boolean outputs using the distribution *Beta*(*α* = 1, *β* = 5). In this context, the loop starts with a dataset of size 64 having all perturbations (and the corresponding artificial measurements) including all combinations of 0 or 1 stimulus with 0 or 1 inhibitor. For the following iterations, optimal sets of signaling perturbations are chosen among all combinations of 0–3 stimuli with 0–2 inhibitors. The maximum number of perturbations to discriminate a given set of behaviors was set to 5, and the maximum number of experiments allowed in the dataset used for learning was set to 80. Additionally, for each gold standard dataset, starting from the same 64 initial datasets, we performed 50 random selections of 10 and 16 experiments. These experiments were added to the initial datasets and we learned BNs from randomly selected experiments. Finally, we computed the testing MSE of this family of BNs with respect to the total 2^14^ experiments.

#### 3.1.2 Real Case Study

To validate this approach on a real phosphoproteomic dataset, we used a larger PKN than in the *in silico* dataset. The reason being that 2 phosphoproteomics datasets were available for this PKN: a low combinatorial one referred to as *screening*, where only 1 stimulus was perturbed per experiment, and the *follow-up*, which had greater combinatorial perturbations. The PKN and datasets were introduced in Melas et al. (2012). The PKN was constructed from several sources of information; it was pruned using the *screening* dataset to keep only the signaling pathways that show a significant response on specific cells. The *follow-up* dataset had 120 combinatorial signaling perturbations and was used to learn optimal BNs fitting the data. The experimental setup for this dataset consisted of 12 stimuli, 3 inhibitors, and 16 readouts. In this context, the loop starts with a dataset having the 12 responsive experiments from the screening dataset. Then, in the following iterations, optimal sets of perturbations are chosen among the available experiments in the follow-up dataset. It is worth noting that at each iteration, there may be several optimal sets of perturbations to discriminate behaviors at hand. Thus, we executed the loop 30 times and at each iteration, one among all optimal sets of signaling perturbations was randomly chosen. The maximum number of perturbations to discriminate a given set of behaviors was set to 5, and the maximum number of experiments allowed in the dataset used for learning was set to 50. Additionally, starting from the same 12 initial datasets, we performed 30 random selections of 20 and 38 experiments. These experiments were added to the initial datasets and we learned BNs from randomly selected experiments. Finally, we computed the testing MSE of this family of BNs with respect to the total 120 experiments.

**Figure 2:**
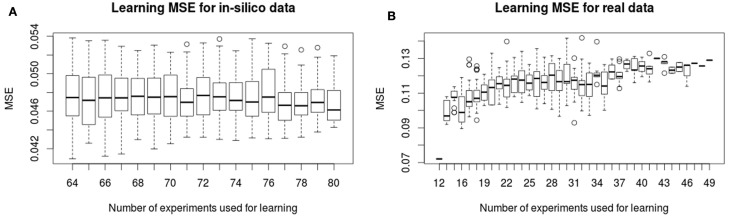
**Learning MSE for *in silico* (A) and real (B) phosphoproteomic datasets**. The *X*-axis shows the number of experiments (optimal signaling perturbations and measurements associated) used for learning at each iteration. The *Y* -axis shows the learning MSE obtained at each iteration, it represents the quality of the learned models with respect to the experiments used in the learning step.

**Figure 3:**
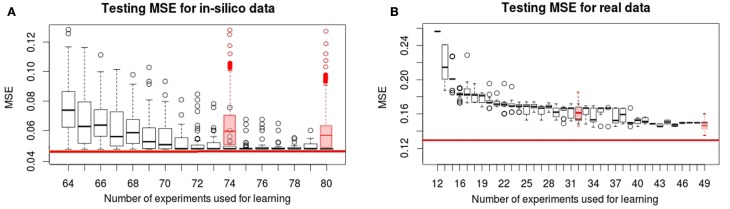
**Testing MSE for *in silico* (A) and real (B) phosphoproteomic datasets**. The red line represents the optimal MSE learned using the full available experimental datasets (2^14^ experiments for *in silico* and 120 for real datasets). The *X*-axis shows the number of experiments (optimal signaling perturbations and measurements associated) used for learning at each iteration. The *Y* -axis shows the testing MSE obtained at each iteration, it represents the quality of the learned models with respect to the full experimental dataset at each iteration. Red boxplots are the results obtained when the set of signaling perturbations was composed of randomly selected experiments of size 74 or 80 for the *in silico* case, and 32 or 49 for the real case.

In Figure 2, we show the evolution of the learning MSE for 100 artificial datasets and the 30 real data executions. In Figure 3, we show the evolution of the testing MSE for the same artificial and real datasets. For the artificial dataset, we observe that the learning MSE remains constant independent from the number of experiments used; while for the real dataset, it slightly increases with the number of experiments. For the real case, we observe a significative difference in the learning MSE of the logic models learned from a low combinatorial set of experiments (screening data) compared to the MSE of those learned from a more combinatorial follow-up dataset (Figure 2B). For both datasets, we observe that the testing MSE converges to the optimal MSE obtained when using the full available datasets. For the artificial case, the testing MSE converges exactly to the optimal MSE (0.047) after 10 experiments; a random selection of experiments is far from reaching this MSE value. For the real case, it converges to an MSE (0.149) at a 15% distance from optimal MSE after 31 experiments; a random selection of experiments shows comparable results. In contrast to a random selection, the proposed method guarantees selecting perturbations that can propose networks with few input–output behaviors: in the real and *in silico* executions, learned logic models had two to eight behaviors after optimal experimental design. One run of the workflow for the artificial case studies proposes 5–16 (on average 11.5) signaling perturbations, while one run of the workflow for real case studies proposes 7–37 (on average 18.9) optimal signaling perturbations. This shows a space of input–output behaviors more difficult to discriminate, therefore requiring more signaling perturbations in the real case study. In 80% of the artificial benchmarks, the loop terminated because all optimal experimental designs were already proposed, in 13%, because the number of 80 allowed experiments was reached, and in 7%, when the search space considered by exploring all size and fitness tolerance range yielded only one input–output behavior. For the real case, in 42% of cases, the loop terminated because all optimal experimental designs were already proposed, in 16%, because behaviors were indistinguishable with the available 120 perturbations, in 6% because the number of 50 allowed experiments was exceeded, and in 36%, because the timeout of 48 h was reached.

### 3.2 Proposing experiments to discriminate input–output behaviors

**Figure 4:**
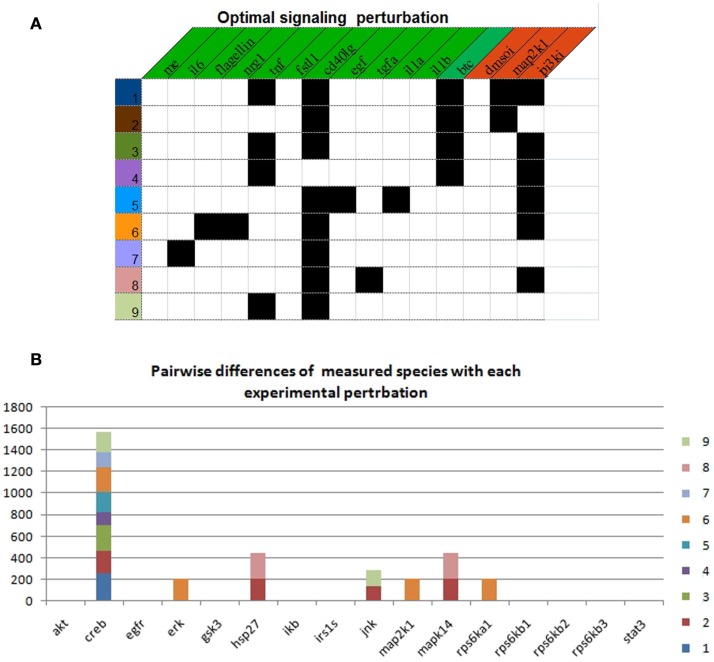
**Optimal experimental design to discriminate between 32 input–output behaviors**. **(A)** Description of each experimental perturbation. Black squares indicate the presence of the corresponding stimulus (green header) or inhibitor (red header). **(B)** Number of pairwise differences by measured species with each experimental perturbation.

Using the real-case PKN and the complete follow-up dataset (120 experiments), we explored the space of nearly optimal BNs by setting 0.2% of tolerance with respect to the minimum MSE. By doing this, we found 35208 BNs describing 32 logical input–output behaviors. Notably, in regards of the available experimental observations and their intrinsic uncertainty, such behaviors explained the data equally well. Next, we identified 6558 optimal sets of signaling perturbations, having 0–3 stimuli combined with 0–2 inhibitors, in order to discriminate among the 32 input–output behaviors. Each optimal set consists of 9 signaling perturbations yielding 3378 pairwise differences. In Figure 4A, we show one example of optimal signaling perturbations. Next, we looked at which specific measured species generated differences (Figure 4B). On the one hand, for one measured species, viz., *CREB*, we generated pairwise differences with eight of the nine proposed signaling perturbations. On the other hand, for all other measured species, we generated pairwise differences with at most two out of the nine signaling perturbations. Moreover, for three measured species, viz., *ERK*, *MAP2K*1, and *rps6Ka*1, we generated pairwise differences with only one experimental perturbation (#7).

## 4 Discussion

The main result of this paper is that the experimental design loop combining learning and discriminating steps shows a fast convergence of the testing MSE (computed with respect to the complete space of signaling perturbations under consideration) to an approximation of its optimal value: in the real case study, based on a very small initial screening dataset (12 perturbations), 30 well-chosen perturbations are enough to learn input–output behaviors whose fitness with respect to the total *follow-up* perturbations is 15% greater than the optimal MSE. This confirms that follow-up phosphoproteomics assays can be highly redundant and should be designed carefully.

### 4.1 Testing MSE non-monotonic evolution

In artificial case studies, the learning MSE (computed with respect to the dataset used for learning) remains somehow constant when new perturbations are added to the dataset. This suggests that the 64 perturbations from the initial dataset may be enough to constrain the training method in a part of the search space, which is close to an optimal Boolean network. Then, introducing sub-optimality searches in the loop allows us to explore efficiently the search space of Boolean networks around such an optimum. On the contrary, the learning MSE for the real case study appears to be very heterogeneous at each iteration of the proposed workflow. The best models optimizing the fitness to the 12 screening data (single stimulus) are at a very small distance (0.07), suggesting that the Boolean networks explaining properly the data should be easy to identify. However, as soon as the observations from additional perturbations (each consisting of a combination of different stimulus and inhibitor species) is added to the dataset, the learning fitness increases to (0.11–0.15) showing a significant variability. This suggests that the best models optimizing each dataset are placed in different parts of the search space and that the training dataset is not robust to small variations. Altogether, values for testing MSE evidence that the discriminative method should be always applied iteratively: after some iterations, it may appear that, although a family of optimal BNs has been totally discriminated, applying a step of discrimination for a closer model to the optimal BNs finally allows to identify BNs with a better fitting. Concretely, our analysis strongly suggests that not only the 120 *follow-up* perturbations are redundant, but also that additional experiments to the 120 at hand are needed to improve the robustness of the BNs identification process. Finally, the validation of the method when using random selection is difficult to evaluate for the real case, since our search space of optimal signaling perturbation was constrained to the 120 available experiments. While in this case the performance was not better than random, we have shown in artificial cases how our method is significantly better than random in larger space of experiments.

### 4.2 Introduction of sub-optimality criteria in the learning step

**Figure 5:**
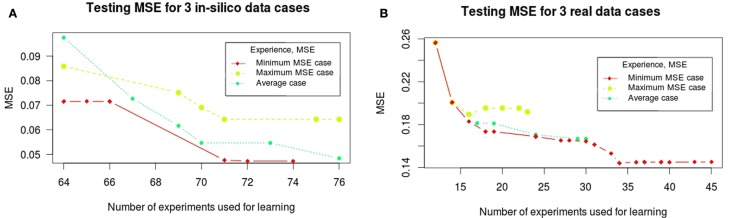
**Trajectories of the testing MSE for three significative cases for *in silico* (A) and real (B) phosphoproteomic datasets: the case where a maximum testing MSE was found, where an average testing MSE was found, and when the minimal testing MSE was found**. The *X*-axis shows the number of experiments (optimal signaling perturbations and measurements associated) used for learning at each iteration. The *Y* -axis shows the testing MSE obtained at each iteration; it represents the quality of the learned models with respect to the full experimental dataset at each iteration.

The above mentioned behavior of the learning MSE confirms that the space of optimal logic models returned by training procedures is very sensitive to the dataset under consideration. That is, it may constantly change when observations of new perturbations are being considered (see the toy example provided in Supplementary Material). Relaxing the tolerance of optimality in our learning procedure allows us in many cases (75% of artificial case studies and 40% of real case studies) to learn new perturbations that will decrease the testing MSE at each iteration (see Figure 5). On the contrary, in other cases, it heavily altered the space of learned logical models yielding a larger MSE. In these cases, however, the MSE of learned logical networks was always improved in a later step. Whereas the cycle of learning and experimental design for artificial case studies follows homogeneous trajectories in all 100 cases (the number of iterations was on average 6.45 with *σ* = 2.3, the number of experiments selected was on average 11.5 with *σ* = 3.2), the cycle for real case studies shows more variability in the 30 considered cases (the number of iterations was on average 10.4 with *σ* = 4.5, the number of experiments selected was on average 18.9 with *σ* = 8.1). In Figure 5, we show the trajectories of this cycle for three significative cases in both datasets: the case where a maximum testing MSE was found, where an average testing MSE was found, and when the minimal testing MSE was found.

### 4.3 Technological constraints

The technological criteria used in this work focused on minimizing the number of perturbed species; however, many other criteria could be taken into account. For example, we could assign weights to stimuli and inhibitors in order to describe the cost of each perturbation and minimize the required budget. Also, if certain stimuli and/or inhibitors are not compatible with each other, we could consider additional constraints in order to avoid such combinations. Finally, a constraint can be added to reduce the *variability* on the selection of inhibitors, since inhibitions require additional control experiments. Adding such criteria may be useful given the fact that we often found many optimal sets of signaling perturbations that would allow to discriminate a family of Boolean networks equally well. In addition, when full pairwise discrimination of input–output behaviors is not possible, we could define an objective function in order to maximize the number of discriminated pairs using a fixed number of perturbations. Interestingly, an important feature of the computational method adopted, that is, Answer Set Programming, is to easily allow for modifications of the constraints over the search space. Thus, the framework that we propose in the tool caspo is intentionally rather generic and should be adapted to the characteristics of the signaling system, which is studied.

## Conflict of Interest Statement

The authors declare that the research was conducted in the absence of any commercial or financial relationships that could be construed as a potential conflict of interest.

## Supplementary Material

The Supplementary Material for this article can be found online at http://journal.frontiersin.org/article/10.3389/fbioe.2015.00131

## Footnotes

^1^http://bioasp.github.io/caspo/

^2^An implementation is publicly available at http://github.com/svidela/sbloopy

## References

[B1] Alexopoulos L. G. Saez-Rodriguez J. Cosgrove B. Lauffenburger D. A. Sorger P. (2010). Networks inferred from biochemical data reveal profound differences in toll-like receptor and inflammatory signaling between normal and transformed hepatocytes. Mol. Cell. Proteomics 9, 1849–1865. 10.1074/mcp.M110.000406 20460255 PMC2938121

[B2] Atias N. Gershenzon M. Labazin K. Sharan R. (2014). Experimental design schemes for learning Boolean network models. Bioinformatics 30, i445–i452. 10.1093/bioinformatics/btu451 25161232 PMC4147904

[B3] Barrett C. L. Palsson B. Ø (2006). Iterative reconstruction of transcriptional regulatory networks: an algorithmic approach. PLoS Comput. Biol. 2:e52. 10.1371/journal.pcbi.0020052 16710450 PMC1463018

[B4] Busetto A. G. Hauser A. Krummenacher G. Sunnåker M. Dimopoulos S. Ong C. S. (2013). Near-optimal experimental design for model selection in systems biology. Bioinformatics 29, 2625–2632. 10.1093/bioinformatics/btt436 23900189 PMC3789540

[B5] Gebser M. Kaminski R. Kaufmann B. Schaub T. (2012). Answer Set Solving in Practice. Synthesis Lectures on Artificial Intelligence and Machine Learning. Morgan and Claypool Publishers.

[B6] Guziolowski C. Videla S. Eduati F. Thiele S. Cokelaer T. Siegel A. (2013). Exhaustively characterizing feasible logic models of a signaling network using answer set programming. Bioinformatics 29, 2320–2326. 10.1093/bioinformatics/btt393 23853063 PMC3753570

[B7] Ideker T. E. Thorsson V. Karp R. M. (2000). “Discovery of regulatory interactions through perturbation: inference and experimental design,” in Pacific Symposium on Biocomputing, Vol. 5, eds Altman R. B. Dunker A. K. Hunter L. Klein T. E. 305–316. 10.1142/9789814447331_0029 10902179

[B8] Kauffman S. (1969). Metabolic stability and epigenesis in randomly constructed genetic nets. J. Theor. Biol. 22, 437–467. 10.1016/0022-5193(69)90015-0 5803332

[B9] Kremling A. Fischer S. Gadkar K. Doyle F. Sauter T. Bullinger E. (2004). A benchmark for methods in reverse engineering and model discrimination: problem formulation and solutions. Genome Res. 14, 1773–1785. 10.1101/gr.1226004 15342560 PMC515324

[B10] Kreutz C. Timmer J. (2009). Systems biology: experimental design. FEBS J. 276, 923–942. 10.1111/j.1742-4658.2008.06843.x 19215298

[B11] Macnamara A. Terfve C. Henriques D. Bernabé B. P. Saez-Rodriguez J. (2012). State-time spectrum of signal transduction logic models. Phys. Biol. 9, 045003. 10.1088/1478-3975/9/4/045003 22871648

[B12] Marler R. T. Arora J. S. (2004). Survey of multi-objective optimization methods for engineering. Struct. Multidiscipl. Optim. 26, 369–395. 10.1007/s00158-003-0368-6

[B13] Mbodj A. Junion G. Brun C. Furlong E. E. Thieffry D. (2013). Logical modelling of *Drosophila* signalling pathways. Mol. Biosyst. 9, 2248–2258. 10.1039/c3mb70187e 23868318

[B14] Melas I. N. Mitsos A. Messinis D. E. Weiss T. S. Saez Rodriguez J. Alexopoulos L. G. (2012). Construction of large signaling pathways using an adaptive perturbation approach with phosphoproteomic data. Mol. Biosyst. 8, 1571–1584. 10.1039/c2mb05482e 22446821

[B15] Mélykúti B. August E. Papachristodoulou A. El-Samad H. (2010). Discriminating between rival biochemical network models: three approaches to optimal experiment design. BMC Syst. Biol. 4:38. 10.1186/1752-0509-4-38 20356406 PMC2873315

[B16] Meyer P. Cokelaer T. Chandran D. Kim K. H. Loh P.-R. Tucker G. (2014). Network topology and parameter estimation: from experimental design methods to gene regulatory network kinetics using a community based approach. BMC Syst. Biol. 8:13. 10.1186/1752-0509-8-13 24507381 PMC3927870

[B17] Mitsos A. Melas I. Siminelakis P. Chairakaki A. Saez-Rodriguez J. Alexopoulos L. G. (2009). Identifying drug effects via pathway alterations using an integer linear programming optimization formulation on phosphoproteomic data. PLoS Comput. Biol. 5:e1000591. 10.1371/journal.pcbi.1000591 19997482 PMC2776985

[B18] Morris M. Saez-Rodriguez J. Sorger P. Lauffenburger D. A. (2010). Logic-based models for the analysis of cell signaling networks. Biochemistry 49, 3216–3224. 10.1021/bi902202q 20225868 PMC2853906

[B19] Remy E. Ruet P. Thieffry D. (2008). Graphic requirements for multistability and attractive cycles in a Boolean dynamical framework. Adv. Appl. Math. 41, 335–350. 10.1016/j.aam.2007.11.00316399639

[B20] Saez-Rodriguez J. Alexopoulos L. G. Epperlein J. Samaga R. Lauffenburger D. A. Klamt S. (2009). Discrete logic modelling as a means to link protein signalling networks with functional analysis of mammalian signal transduction. Mol. Syst. Biol. 5, 331. 10.1038/msb.2009.87 19953085 PMC2824489

[B21] Shannon C. E. (1948). A mathematical theory of communication. Bell Syst. Tech. J. 27, 379–423. 10.1002/j.1538-7305.1948.tb00917.x

[B22] Sharan R. Karp R. M. (2013). Reconstructing Boolean models of signaling. J. Comput. Biol. 20, 249–257. 10.1089/cmb.2012.0241 23286509 PMC3590894

[B23] Sparkes A. Aubrey W. Byrne E. Clare A. Khan M. N. Liakata M. (2010). Towards robot scientists for autonomous scientific discovery. Autom. Exp. 2, 1. 10.1186/1759-4499-2-1 20119518 PMC2813846

[B24] Stegmaier J. Skanda D. Lebiedz D. (2013). Robust optimal design of experiments for model discrimination using an interactive software tool. PLoS ONE 8:e55723. 10.1371/journal.pone.0055723 23390549 PMC3563641

[B25] Szczurek E. Gat-Viks I. Tiuryn J. Vingron M. (2008). Elucidating regulatory mechanisms downstream of a signaling pathway using informative experiments. Mol. Syst. Biol. 5, 287–287. 10.1038/msb.2009.45 19584836 PMC2724975

[B26] Vatcheva I. de Jong H. Bernard O. Mars N. J. I. (2005). Experiment selection for the discrimination of semi-quantitative models of dynamical systems. Artif. Intell. 170, 472–506. 10.1016/j.artint.2005.11.001

[B27] Videla S. Guziolowski C. Eduati F. Thiele S. Gebser M. Nicolas J. (2014). Learning Boolean logic models of signaling networks with ASP. Theor. Comput. Sci. 10.1016/j.tcs.2014.06.022

[B28] Yeang C. Mak H. C. McCuine S. Workman C. Jaakkola T. Ideker T. E. (2005). Validation and refinement of gene-regulatory pathways on a network of physical interactions. Genome Biol. 6, R62. 10.1186/gb-2005-6-7-r62 15998451 PMC1175993

