# Supplementary material for: Designing Experiments to Discriminate Families of Logic Models
Source: Front Bioeng Biotechnol. 2015 Sep 4;3:131. doi: 10.3389/fbioe.2015.00131 (PMC4560026; doi:10.3389/fbioe.2015.00131)
Supplement: Supplementary file 1 [file data_sheet_1.pdf]

Supplementary Material: Designing experiments to discriminate families of logic models

Santiago Videla[1,2,3,4], Irina Konokotina[7], Leonidas G Alexopoulos[5], Julio Saez-Rodriguez[6], Torsten Schaub[3], Anne Siegel[1,2], Carito Guziolowski[7,*]

[1] *CNRS, UMR 6074 IRISA, Campus de Beaulieu, Rennes, France*
[2] *INRIA, Dyliss project, Campus de Beaulieu, Rennes, France*
[3] *Universität Potsdam, Institut für Informatik, Potsdam, Germany*
[4] *LBSI, Fundación Instituto Leloir - CONICET, Buenos Aires, Argentina*
[5] *Department of Mechanical Engineering, NTU, Athens, Greece*
[6] *European Molecular Biology Laboratory, European Bioinformatics Institute, Hinxton, UK*
[7] *IRCCyN UMR CNRS 6597, École Centrale de Nantes, Nantes, France*

Correspondence*:
Carito Guziolowski
IRCCyN UMR CNRS 6597, École Centrale de Nantes, 1 rue de la Noë, Nantes, 44321, France, carito.guziolowski@irccyn.ec-nantes.fr

1 SUPPLEMENTARY ALGORITHMS

In what follows we provide pseudo-code algorithms describing the workflow used for testing our experimental design method (Figure 1). First, let us introduce the required notation for such algorithms. We denote a prior knowledge network with nodes V and edges E with (V, E). We use (P, O) to denote a phospho-proteomics dataset consisting of P signaling perturbations and their corresponding vector observations $O : P \to [0, 1]^m$, where m is the number of readouts. In particular, we use (Ψ, Θ) to denote the dataset under consideration for testing purposes. On the one hand, for artificial case studies (Ψ, Θ) corresponds to the complete simulated dataset with respect to certain *gold standard*. On the other hand, for real case studies (Ψ, Θ) corresponds to the available follow-up dataset. Notably, in a real-life application, one would have only the list of feasible signaling perturbations Ψ, while observations will be generated after performing concrete wet experiments.

We assume the existence of a function $LEARN$ for learning nearly optimal BNs and their corresponding set of input-output behaviors. Such a function is parametrized by the PKN (V, E), the phospho-proteomics dataset (P, O), $F \in [0, 1]$ and $S \geq 0$ specifying allowed tolerances with respect to optimal fitness and size, respectively. In addition, we assume a function MSE which returns the Mean Squared Error for a set of behaviors $\boldsymbol{B}$ with respect to a given phospho-proteomics dataset (P, O). Also, we assume the existence of a function $DISCRIMINATE$ implementing the method described in Section 2.2. In this case, the parameters are the set of input-output beahaviors $\boldsymbol{B}$, the list of feasible perturbations Ψ, the maximum allowed number of stimuli s, inhibitors i, and perturbations k to discriminate the given set of behaviors, and the Boolean flag $relax$ specifying whether we require full pairwise discrimination or not. As a results, $DISCRIMINATE$ returns all optimal sets of signaling perturbations to discriminate behaviors in $\boldsymbol{B}$ according to the given parameters. Note that there may be no solutions, i.e., an empty set is returned.

Algorithm S1 shows the pseudo-code for an auxiliary function implementing the execution of "dry experiments". The inputs of such a function are the dataset (P, O), sets of desired signaling perturbations $\mathcal{D}$, and the available observations Θ. The goal of this function is to extend the dataset (P, O) with a randomly chosen set of perturbations $\boldsymbol{D} \in \mathcal{D}$ by taking the corresponding observations from Θ. In addition, the function ensures that the total number of experiments in (P, O) remains lower or equal a global value $MAX\_EXPERIMENTS\_ALLOWED$. If there exists a set of perturbations in $\mathcal{D}$ to extend the current dataset (P, O), the function returns the value $\boldsymbol{True}$ and (P, O) extended accordingly. Otherwise, the function returns the value $\boldsymbol{False}$ and (P, O) unchanged. It is worth noting that for the given pseudo-code we rely on the *restriction* and *union* of functions. Thus, let us recall the corresponding definitions for each case. For $f : X \rightarrow Y$ and $S \subseteq X$, the *restriction* of f to S is the function $f|_S : S \rightarrow Y$ such that $f|_S(s) = f(s)$ for all $s \in S$. Further, the *union* of $f : X \rightarrow Y$ and $g : W \rightarrow Y$ with $X \cap W = \emptyset$ is denoted as $(f \cup g) : (X \cup W) \rightarrow Y$ such that $(f \cup g)(z) = f(z)$ if $z \in X$ and $(f \cup g)(z) = g(z)$ if $z \in W$.

Algorithm S1. Perform dry experiments

function DRY_EXP((P, O): current dataset, $\mathcal{D}$: sets of perturbations, Θ: available observations)
  while $\mathcal{D} \neq \emptyset$ **do**
    $\boldsymbol{D} \leftarrow random\_pop(\mathcal{D})$ ▷ a random $\boldsymbol{D}$ is returned and removed from $\mathcal{D}$
    $\boldsymbol{D} \leftarrow \boldsymbol{D} \setminus P$ ▷ keep only perturbations not included in P already
    if $\boldsymbol{D} \neq \emptyset$ **then**
      if $|\boldsymbol{D}| + |P| \leq MAX\_EXPERIMENTS\_ALLOWED$ **then**
        $(P, O) \leftarrow (P \cup \boldsymbol{D}, O \cup \Theta|_{\boldsymbol{D}})$ ▷ Extends (P, O) using the restriction of Θ to $\boldsymbol{D}$
        return $\boldsymbol{True}, (P, O)$
      else
        return $\boldsymbol{False}, (P, O)$ ▷ Number of allowed experiments reached
      end if
    end if
  end while
  return $\boldsymbol{False}, (P, O)$ ▷ All sets of perturbations were performed already, i.e., $\boldsymbol{D} \subseteq P, \forall \boldsymbol{D} \in \mathcal{D}$
end function

Algorithm S2 shows the pseudo-code for the main function implementing our workflow. Line 3 starts the main loop controlled with the Boolean variable *done*. In Line 6 the algorithm calls the function $LEARN$ in order to find all strictly optimal input-output behaviors, i.e., no tolerance on neither fitness nor size. Then, in Line 8 and Line 9 the algorithm prints the *learning* and *testing* MSE respectively. Notably, in a real-life application the *testing* MSE can not be computed as we would not have access to Θ. Next, if only 1 input-output behavior is found, in Line 12 to Line 21, the algorithm explores nearly optimal behaviors by considering a range of tolerances, first over the size and then over the fitness. After exploring nearly optimal models, if there is still only 1 input-output behavior, the algorithm goes to Line 41 and terminates the loop. Otherwise, in Line 26 the algorithm calls the function $DISCRIMINATE$ in order to discriminate among found behaviors requiring full pairwise discrimination. If there is at least one solution, i.e., an optimal set of perturbations, the algorithm calls the auxiliary function DRY_EXP and stores the returned value overriding variables $done$ and (P, O). If $done$ is set to $\boldsymbol{False}$ (i.e., $extended$ is set to $\boldsymbol{True}$), then it means that (P, O) was extended with new perturbations and their corresponding observations. Thus, the loop will start over from Line 4. On the contrary, if $done$ is set to $\boldsymbol{True}$ (i.e., $extended$ is set to $\boldsymbol{False}$), then it means that (P, O) could not be extended and the loop terminates. In addition, in Line 26 the call to $DISCRIMINATE$ may fail due to the requirement of full pairwise discrimination and return an empty set. In such a case, in Line 32 the algorithm calls the function $DISCRIMINATE$ one more time but without requiring full pairwise discrimination. If there is at least one solution the algorithm proceeds as before. Otherwise, it means that it is not possible to generate any

difference among the behaviors at hand considering the given list of feasible perturbations. Thus, *done* is set to $\boldsymbol{True}$ and the loop terminates.

Algorithm S2. Loop for learning and discriminating input-output behaviors

```
function WORKFLOW((V, E): PKN, (P, O): initial dataset, (Ψ, Θ): testing dataset, (s, i, k): maximum
    number of stimuli, inhibitors, and perturbations to discriminate a given set of behaviors)
    done ← False
    while not done do
        F ← 0
        S ← 0
        B ← LEARN((V, E), (P, O), F, S)                    ▷ Learning without tolerance

        print MSE(B, (P, O))                 ▷ Learning MSE, i.e. with respect to (P, O)
        print MSE(B, (Ψ, Θ))                 ▷ Testing MSE, i.e. with respect to (Ψ, Θ)

        if |B| = 1 then
            while |B| = 1 and S ≤ 5 do
                S ← S + 1
                B ← LEARN((V, E), (P, O), 0, S)       ▷ Learning with size tolerance S
            end while
            if |B| = 1 then
                while |B| = 1 and F ≤ 0.05 do
                    F ← F + 0.01
                    B ← LEARN((V, E), (P, O), F, 0)    ▷ Learning with fitness tolerance F
                end while
            end if
        end if

        if |B| > 1 then
            relax ← False
            D ← DISCRIMINATE(B, Ψ, s, i, k, relax)
            if D ≠ ∅ then
                extended, (P, O) ← DRY_EXP((P, O), Θ, D)
                done ← not extended
            else
                relax ← True              ▷ Cannot discriminate all behaviors pairwise
                D ← DISCRIMINATE(B, Ψ, s, i, k, relax)
                if D ≠ ∅ then
                    extended, (P, O) ← DRY_EXP((P, O), Θ, D)
                    done ← not extended
                else
                    done ← True    ▷ Cannot generate any difference among given behaviors
                end if
            end if
        else
            done ← True    ▷ only 1 input-output behavior after looking at nearly optimal models
        end if
    end while
end function
```

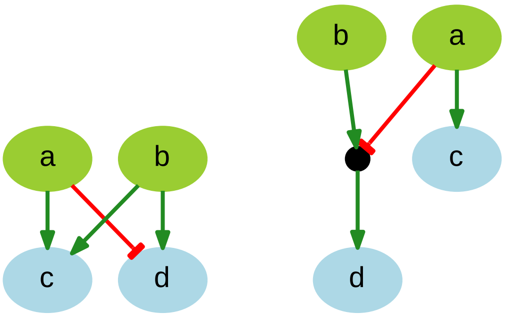

(a) (b)

	Stimuli		Readouts		
#	a	b	c	d	used at ...
1	0	1	0	1	Iteration 0
2	1	0	1	0	Iteration 0
3	0	0	0	0	Iteration 1
4	1	1	1	0	Iteration 2

(c)

Figure S1. Toy example to illustrate the learning loop. Green and red arrows refer to activations and inhibitions respectively. Green nodes are (possible) stimuli while blue nodes are readouts or measured species. (a) A toy prior knowledge network (PKN). (b) A gold standard BN derived from the PKN shown in (a). (c) An artificial dataset of all possible perturbation and their corresponding responses over the gold standard shown in (b).

2 TOY EXAMPLE ILLUSTRATION FOR THE EXPERIMENTAL DESIGN LOOP

An important result from our work is the fact that we need to introduce tolerances in the learning loop because when learning only strictly optimal models with respect to a partial experimental dataset, we may miss (global) optimal models with respect to the complete experimental dataset. Let us illustrate this with the following toy example.

We consider a PKN (Figure S1a) in which a and b are stimuli while c and d, are readouts. From this PKN we derived a gold standard Boolean network shown in Figure S1b. Then, for the given experimental setup, all possible perturbations and their corresponding responses over the gold standard are shown in Figure S1c. Next, we start the learning loop with the PKN (Figure S1a) and using experiments #1 and #2 (Figure S1c). The optimal BNs learned (without tolerance) will be a family of two BNs (Iteration 0 in Figure S2), which has 2 input-output behaviors. If we compute the optimal signaling perturbations to discriminate such behaviors, the experiment proposed is #3 in Figure S1c ($a = 0$, $b = 0$). Once the experiment #3 is added to the dataset used for learning, the resulting optimal models is actually a single BN (Iteration 1 (top) in Figure S2). Notably, with 1 BN there can be only one input-output behavior. Thus, our method aims at learning nearly optimal BNs considering a range of tolerances with respect to the optimal size and fitness. In our toy example, considering BNs with size larger than the optimal by 1, yields a family of two BNs (Iteration 1 (bottom) in Figure S2), describing 2 input-output behaviors. The optimal signaling perturbation proposed to discriminate these input-output behaviors is the experiment #4 in Figure S1c ($a = 1$, $b = 1$). Finally, learning optimal BNs using experiments #1 to #4 yields the gold standard BN (Iteration 2 in Figure S2). It is worth noting that the input-output behaviors in all the steps are not necessarily the same, since BN topologies appear and dissapear from the set of optimal BNs compatible as the dataset changes. Importantly, without the incremental procedure of tolerance parameters, we would have ended the learning loop at iteration 1 and without finding the gold standard.

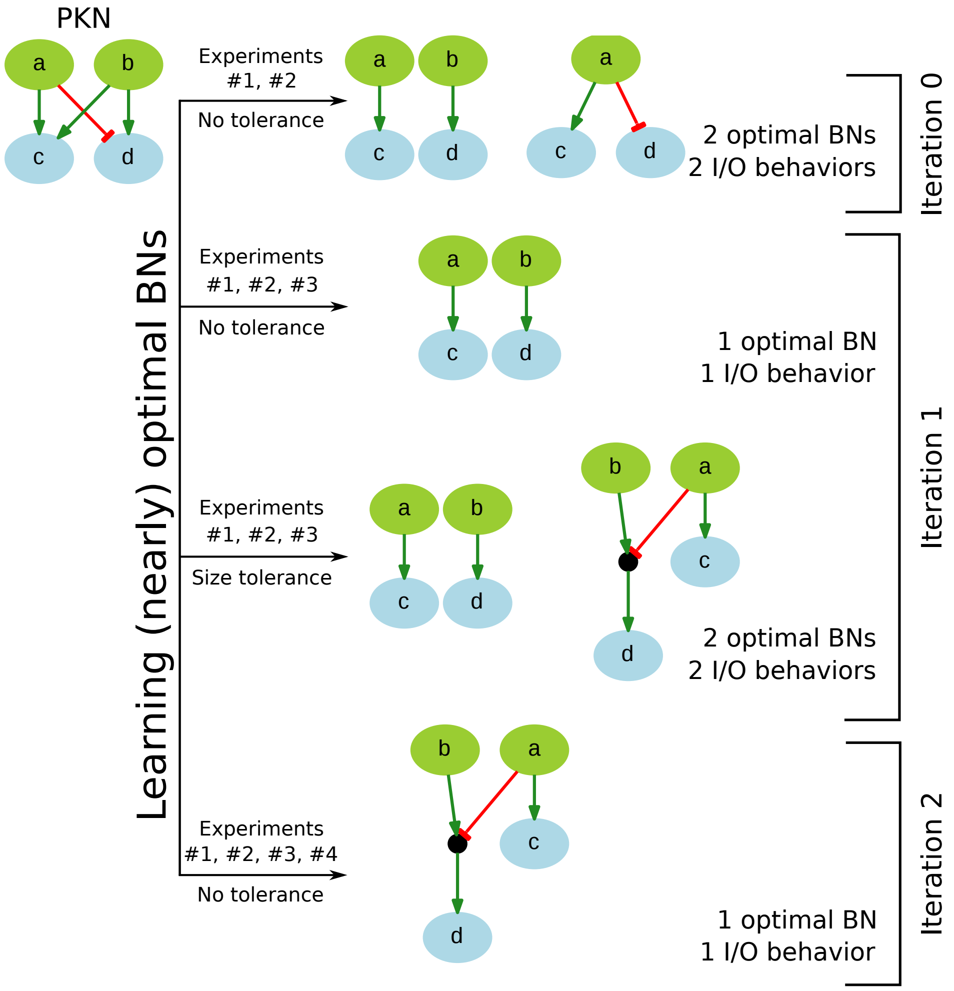

Figure S2. Toy example to illustrate the learning loop as new experiments are added to the used dataset. At each iteration BNs are learned from the PKN and artificial experimental data shown in Figure S1c. Iteration 0 uses experiments #1 and #2 without tolerance. Iteration 1 uses experiments #1, #2, and #3 with (bottom) and without (top) tolerance. Iteration 2 uses experiments #1, #2, #3, and #4 without tolerance which yields the gold standard BN used to generate the dataset.
